# Incidence of Pediatric Perforated Appendicitis during the COVID-19 Pandemic; a Systematic Review and Meta-Analysis

**DOI:** 10.22037/aaem.v10i1.1421

**Published:** 2021-01-01

**Authors:** Gholamreza Motazedian, Poorya Aryanpoor, Ehsan Rahmanian, Samaneh Abiri, Navid Kalani, Naser Hatami, Farhad Bagherian, Mohammad Etezadpour, Roohie Farzaneh, Fatemeh Maleki, Mahdi Foroughian, Mojtaba Ghaedi

**Affiliations:** 1 Plastic & Reconstructive Surgery Department, Shiraz University of Medical Sciences, Shiraz, Iran.; 2 Student Research Committee, Jahrom University of Medical Sciences, Jahrom, Iran.; 3 Research center for social Determinants of Health, Jahrom University of Medical Sciences, Jahrom, Iran.; 4 Department of Emergency Medicine, Babol University of Medical Sciences, Babol, Iran.; 5 Surgery department, Faculty of Medicine, Mashhad University of Medical sciences, Mashhad, Iran.; 6 Department of Emergency Medicine, Faculty of Medicine, Mashhad University of Medical Sciences, Mashhad, Iran.; 7 Department of Emergency Medicine, Faculty of Medicine, Birjand University of Medical Sciences, Birjand, Iran.; 8 Plastic Surgery department, Jahrom University of Medical Sciences, Jahrom, Iran.

**Keywords:** COVID-19, Appendicitis, Ruptured, Abdomen, Acute

## Abstract

**Introduction:**

COVID-19 has affected the pattern of referral to medical centers and quarantine against COVID-19 might delay referral and management of surgical emergencies. This study aimed to compare the pooled event rate of pediatric perforated appendicitis before and during the COVID-19 pandemic.

**Methods:**

This was a systematic review and meta-analysis study based on the PRISMA guidelines. Scopus, Web of Sciences, and PubMed databases were searched for studies reporting the rate of perforated appendicitis based on the post-appendectomy observations or imaging methods. The Egger bias test and funnel plot were used to detect and depict publication bias. Statistical analysis was performed in Comprehensive Meta-analysis package version 3.

**Results:**

Twelve studies were eligible for inclusion in our study. The pooled prevalence of pediatric perforated appendicitis in the pre-COVID era was 28.5% (CI95%: 28.3 to 28.7%) with a heterogeneity of 99%. In the COVID era, the event rate proportion was 39.4% (CI95%: 36.6 to 42.3%) with a heterogeneity of 99%. There was a significant difference in the subgroup analysis within the pre-COVID and COVID era (P<0.001), showing a higher perforation rate in the COVID era.

**Conclusion:**

Our study showed that during the COVID-19 pandemic, the rate of perforated appendicitis has significantly increased in comparison to before the COVID-19 pandemic.

## 1. Introduction

Diagnosis and decision-making about some diseases like abdominal pain can be challenging for physicians (1). In ‎surgery, the most common cause of abdominal surgery is acute appendicitis. Appendicitis is a surgical emergency and a common disease that can ‎present with a variety of symptoms (2). The manifestations of this disease overlap with several other medical conditions. Sometimes the complexities and ‎differences in the way it occurs can lead even the most experienced physicians to mistake it for other conditions (3). In some cases, especially in children, the symptoms may be deceptive and difficult to diagnose, and on the ‎other hand, prolongation of the disease may be life-threatening or cause severe complications (4). Therefore, ‎correct and timely diagnosis of the disease requires experience and special skills. Lack of ability of children to give an accurate history, delayed diagnosis by parents and physicians, and the ‎presence of nonspecific gastrointestinal disorders are other causes of late diagnosis of acute appendicitis ‎in children (5, 6). Appendicitis in pediatric cases is more likely to rupture, and due to the lack of omentum growth in children, ‎peritonitis is more likely to occur and complications are more common (7). In children under 5 years of age, ‎both the diagnosis of acute appendicitis and the risk of appendix rupture is higher (8). Currently, since COVID-19 has emerged as a pandemic all around the world, some concerns have been raised about the management of surgical emergencies in pediatric cases. COVID-19 has a variety of clinical manifestations, including abdominal pain, which can make it difficult for ‎physicians to differentiate acute appendicitis, and as a result, the risk of perforation of the appendix ‎increases (9). Additionally, the quarantine imposed during the COVID-19 epidemic, as well as the refusal of ‎parents and children to visit hospitals out of fear of getting infected with the virus, could have resulted in the increased rate of perforated ‎appendicitis or its complications. This study aimed to compare the pooled event rate of pediatric perforated appendicitis before and during the COVID-19 pandemic. 

## 2. Methods

***2.1 Study design and setting***

This systematic review and meta-analysis study was done based on the Preferred Reporting Items for Systematic Reviews and Meta-analyses (PRISMA) guidelines. We quarried Scopus, Web of Sciences, and PubMed databases for studies irrespective of the study time, limiting results to English articles, cross-sectional and retrospective or prospective studies using the keywords of Appendicitis, Acute Appendicitis, Perforated, Perforation, Appendix, Appendectomy, Children, and Pediatric. The following search strategy was used “(COVID-19[MeSH Major Topic]) AND (Acute Abdomen [MeSH Major Topic]) AND (Appendicitis [Title])) OR (Acute Appendicitis [Title])) OR (Appendectomy [Title])) OR (Perforated [Title])) OR (Perforation [Title]) AND (all child [Filter])” in PubMed. Scopus search strategy was “#1 - TITLE-ABS-KEY (Appendicitis OR Acute Appendicitis OR Appendectomy OR Perforation) #2 - TITLE-ABS-KEY (COVID-19 OR SARS-Cov2 OR Coronavirus) #3 - Pediatric OR children #4 - #1 AND #2 AND #3” and the same with TS function in Web of sciences. 

Studies reporting the rate of perforated appendicitis based on the post-appendectomy observations or imaging methods were considered eligible to be included in our study if reporting them in the pediatric age group (under 18 years old). 

***2.2 Quality assessment***

The Newcastle-Ottawa Quality Assessment Scale (NOS), which was adapted to measure characteristics of quality relevant to population-based studies of incidence, was used to assess the studies' quality (10). Studies having a low possibility of bias were included. 

***2.3 Measured outcome***

Our study outcome was comparing the proportion of perforated appendicitis within two time periods of the Post COVID-19 era (from December 2019) and pre COVID-19 era (before December 2019). Studies were included from 1995 to 2021. 

***2.4 Statistical analysis***

The Cochran Q test (two-test for heterogeneity) was used to assess the heterogeneity of the studies. I^2 ^and its 95% confidence interval (CI) were used to calculate the percentage of total heterogeneity to total variability. A Q test with a P<0.1 or an I^2^ greater than 60% was considered to show significant statistical heterogeneity.  Random-effects models with double arcsine transformations were used. A 2-sided P<05 was considered statistically significant, regardless of the I^2^ statistic. In order to assess the significant difference in proportions between groups in each analysis, we calculated the inter-group P-value; when there was a significant difference, we ran pairwise comparisons and adjusted the level when necessary. A funnel plot was used to depict publication bias. The Egger bias test and the Begg-Mazumdar rank correlation Kendall 2 statistic were used to detect asymmetry. Statistical analysis was performed in Comprehensive Meta-analysis package version 3 and bias possibility was visualized using Review Manager Version 5.4.1. 

## 3. Results

**Figure 1:**
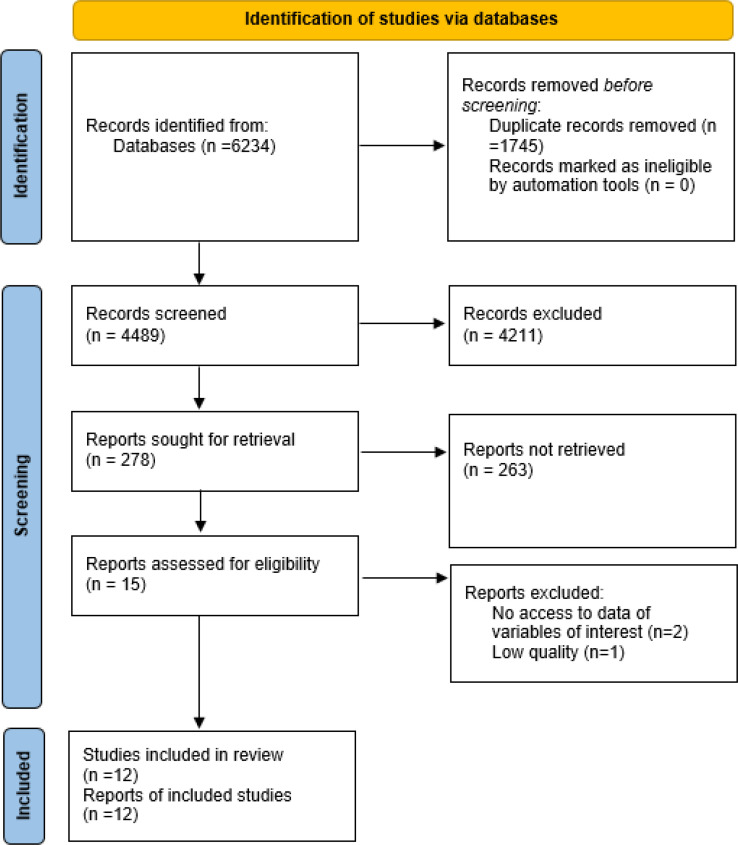
PRISMA flow chart of the present study

As shown in figure 1, there were 6234 articles found in the initial search, conducted by two independent researchers, from which 1745 duplicated articles were removed. Then based on the abstracts, unrelated articles, including review articles, interventional and case-control studies, and studies only on the adult population were excluded from the study. Also, articles whose abstract or main text was not available were excluded. Finally, 278 potentially related articles were listed for full text review. 

Twelve studies were eligible to be included in our study. A checklist including the name of the researcher, article title, year and place of study, sample size, and prevalence of perforated appendicitis was used to collect data (table 1). 8 studies were conducted in COVID era, also containing datasets of before COVID. 9 studies were performed in US, one in Pakistan, one in Germany, and one in UK. The largest sample size belonged to Cheong LH et al. study with 120117 participants. 

***3.1 Prevalence of pediatric perforated appendicitis***

**Figure 2:**
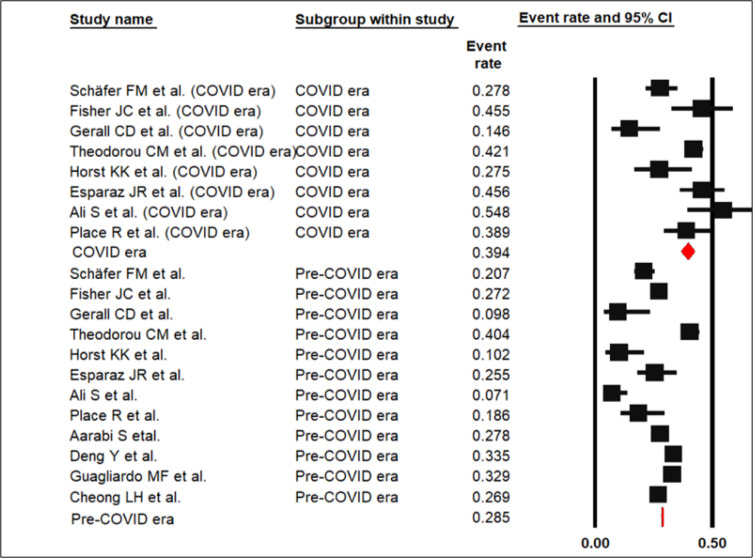
Forest plot of pooled prevalence of perforated appendicitis in pre-COVID and Post-COVID eras (p < 0.001)

The pooled prevalence of pediatric perforated appendicitis in the pre-COVID era was 28.5% (CI95%: 28.3 to 28.7%) with heterogeneity of 99%. In the COVID era event rate proportion was 39.4% (CI95%: 36.6 to 42.3%) with heterogeneity of 99% (p < 0.001; figure 2).

**Figure 3:**
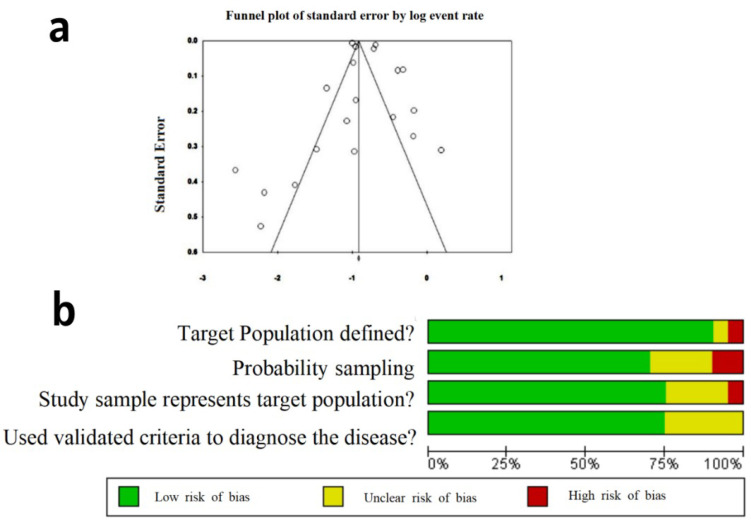
Funnel plot of the study and risk of bias. (a) Funnel plot of the study; (b) risk of bias in the included studies based on the modified Newcastle-Ottawa Quality Assessment Scale (NOS)

Egger's regression revealed no possibility of publication bias (P-2tailed = 0.458), which was in line with the result of Begg and Mazumdar rank correlation (kendall's rau=-0.263, P=0.104; figure 3a). The risk of bias in studies and quality of studies, assessed using a modified NOS, are presented in figure 3b. Most studies showed a low risk of bias. 

## 4. Discussion

Our study showed that there has been an increase in the rate of perforated appendicitis during the COVID-19 pandemic in comparison to before the COVID-19 pandemic

Difficulty diagnosing ‎the disease usually delays the start of treatment and in many cases leads to perforation. While delayed referral to a medical center could also be a potential factor leading to perforation; COVID-19 pandemic has decreased public referral to hospitals due to fear of infection (22). Due to the high ‎mortality rate, appendicitis is on the list of important diseases in children. Considering the rapid progression of ‎this disease in children, the need for timely diagnosis of the disease in order to prevent side effects is ‎emphasized by all surgeons. Despite the existence of radiological techniques for diagnosing appendicitis, it ‎is still difficult to diagnose acute appendicitis. Additionally, physicians should also be aware of COVID-19’s gastrointestinal symptoms that might mimic acute abdomen symptoms (23, 24). In case of ruptured appendicitis, the risk of complications increases. Imposing unnecessary surgery can also cause complications ‎such as intestinal adhesions (25). ‎ Our study indicated that during the COVID-19 pandemic, the rate of perforated appendicitis has significantly increased in comparison to before the COVID-19 pandemic. Additionally, other studies have shown that even before the pandemic, there were seasonal variations in acute appendicitis cases (26) and racial factors might also be affecting the disease prevalence (8). Research has shown that even in one country, the prevalence of acute appendicitis might change over the years (27). Following the outbreak of COVID-19 and the responsibility of hospitals to provide services to patients with COVID-19, the provision of medical services to other patients was largely shut down and only emergencies continued; that might have led to missed cases of some urgent diseases including acute abdomen. These patients will suffer if left untreated. Therefore, hospitals must be prepared to accept non-COVID-19 patients. 

Based on Ojetti et al. study, this issue is not limited to appendicitis. The sharp drop in ED visits during the pandemic might be attributed to fear of the virus, implying that individuals with significant conditions did not seek medical help. Worrying statistics emerged, in particular, about a decrease in cardiology and neurology admissions. Those patients put off seeking medical help out of fear of being exposed to COVID-19, resulting in increased morbidity and mortality (28). We recommend Global community training for dealing with emergencies during the pandemic so that time-critical medical situations are not neglected.

**Table 1:** Characteristics of included studies

**Authors**	**N**	**Perforated**	**Date**	**Study interval time**	**Country**
Schäfer FM et al. (11)	338	70	2021	March 2018 to May 2019	Germany (Bavaria)
Fisher JC et al.(12)	1291	351	2021	Jan 2014 to June 2019	US
Gerall CD et al. (13)	41	4	2021	March to May 2019	US / New York city
Theodorou CM et al. (14)	592	239	2021	Pre-COVID era	US /California
Horst KK et al. (15)	59	6	2021	March 1 to May 31 2019	US /Rochester, Minnesota
Esparaz JR et al. (16)	102	26	2021	March to May 2019	US /Alabama
Ali S et al. (17)	112	8	2020	March to May 2019	Pakistan/Peshawar
Place R et al. (18)	70	13	2020	March 16 to June 7 2019	US/ Northern Virginia
Aarabi S et al. (19)	19019	5282	2011	2000 to 2006	New England
Deng Y et al. (20)	31457	10524	2010	1997	US
Guagliardo MF et al. (8)	9069	2986	2003	1995 and 1997?	US / New York and California
Cheong LH et al. (21)	120117	32321	2014	2004 to 2010	US and Canada
Schäfer FM et al. (11) b	176	49	2021	2020	Germany (Bavaria)
Fisher JC et al. (12)b	55	25	2021	Jan and May 2020	US
Gerall CD et al. (13)b	48	7	2021	March to May 2020	US / New York city
Theodorou CM et al. (14) b	606	255	2021	2019 vs 2020	US /California
Horst KK et al. (15)b	51	14	2021	March 1 to May 31 2020	US / Rochester, Minnesota
Esparaz JR et al. (16)b	103	47	2021	March to May 2020	US /Alabama
Ali S et al. (17)b	42	23	2021	March to May 2020	Pakistan/Peshawar
Place R et al. (18) b	90	35	2021	March 16 to June 7 2020	US/ Northern Virginia

N: number. b, same study containing cohorts of both Pre-COVID and post-COVID eras.

## 5. Limitations

‎There are many factors affecting the prevalence of perforated appendicitis, such as ethnicity, seasonal variation, and the medical centers’ approaches to acute abdomen, which were not considered in our study. 

## 6. Conclusion

Our study showed that the COVID-19 pandemic might have led to an increase in the rate of perforated appendicitis in comparison to the before COVID-19 pandemic. One of the serious health concerns during the pandemic is the state of people's health due to urgent non-COVID-19 diseases, which covers a wide range of patients including acute abdomen cases. 

## 7. Declarations

### 7.1 Acknowledgment

We would like to thank the Clinical Research Development Unit of Peymanieh Educational and Research and Therapeutic Center of Jahrom University of Medical Sciences for pro-viding facilities for this work.

### 7.2 Conflict of interest

The authors have declared that no competing interests exist.

### 7.3 Author contribution

 GM, MG, NK, MF, and FB conceptualized the study questions and performed revisions. PA, NH, and ER performed the searches. GM, MG, SA, ME, FM, and RF conducted the statistical analyses. Other authors provided the draft of the manuscript.

### 7.4 Funding

This research did not receive any grant from funding agencies in the public, commercial, or non-profit sectors.

### 7.5Ethical considerations

All ethical principles were considered in this article.
